# Hidden Node Detection between Observable Nodes Based on Bayesian Clustering

**DOI:** 10.3390/e21010032

**Published:** 2019-01-07

**Authors:** Keisuke Yamazaki, Yoichi Motomura

**Affiliations:** AI Research Center, National Institute of Advanced Industrial Science Technology, 2-4-7 Aomi, Koto-ku, Tokyo 135-0064, Japan

**Keywords:** Bayesian clustering, structure learning in singular cases, model selection

## Abstract

Structure learning is one of the main concerns in studies of Bayesian networks. In the present paper, we consider networks consisting of both observable and hidden nodes, and propose a method to investigate the existence of a hidden node between observable nodes, where all nodes are discrete. This corresponds to the model selection problem between the networks with and without the middle hidden node. When the network includes a hidden node, it has been known that there are singularities in the parameter space, and the Fisher information matrix is not positive definite. Then, the many conventional criteria for structure learning based on the Laplace approximation do not work. The proposed method is based on Bayesian clustering, and its asymptotic property justifies the result; the redundant labels are eliminated and the simplest structure is detected even if there are singularities.

## 1. Introduction

In learning Bayesian networks, one of the main concerns is structure learning. Many criteria to detect the network structure have been proposed such as the minimum description length (MDL) [1], the Bayesian information criterion (BIC) [2], the Akaike information criterion (AIC) [3], and the marginal likelihood [4]. Most of these criteria assume statistical regularity, which means that the network has identifiability on the parameter and then the nodes are observable.

The nodes of the network are not always observable in practical situations; there will be some underlying factors, which are difficult to observe and do not appear in the given data. In such cases, the criteria for the structure learning must be designed by taking account of the existence of the hidden nodes. However, the statistical regularity does not hold when the network contains hidden nodes [5,6].

The probabilistic models fall into two types: Regular and singular. If the parameter and the probability function expressed by the parameter have one-to-one mapping, the model has statistical regularity and is referred to as regular. Otherwise, there are singularities in the parameter space and the model is referred to as singular. Due to the singularities, the Fisher information matrix is not positive definite, which means that the conventional analysis based on the Laplace approximation or the asymptotic normality does not work in the singular models. Many probabilistic models such as mixture models, hidden Markov models, and neural networks are singular. To cope with the problem of the singularities, an analysis method based on algebraic geometry has been proposed [7], and asymptotic properties of the generalization performance and of the marginal likelihood have been investigated in mixture models [8], hidden Markov models [9], neural networks [7,10], etc.

It is known that the Bayesian network with hidden nodes is singular since the parametrization will change compared with the network without hidden nodes. Even in the simple structure such as the naive Bayesian network, the parameter space has singularities [5,11]. A method to select the optimal structure from some candidate networks has been proposed by using the algebraic geometrical method [5]. For general singular models, new criteria are developed; a widely applicable information criterion (WAIC) is based on the asymptotic form of the generalization error and a widely applicable Bayesian information criterion (WBIC) is derived from the asymptotic form of the marginal likelihood. BIC is also extended to the singular models [12].

**Figure 1:**
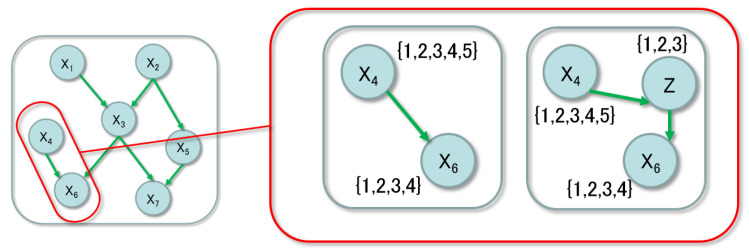
The two-step method: structure learning with observable nodes and hidden-node detection.

The structure learning of the Bayesian network with hidden nodes is a very widely studied problem. Observable constraints from the Bayesian network with hidden nodes is considered in [13]. A model based on observable conditional independence constraints is proposed by [14]. For causal discovery, the related fast causal inference (FCI) algorithm has been developed, e.g., [15]. In the present paper, we consider a two-step method; the first step obtains the optimal structure with observable nodes and the second step detects the hidden nodes in each partial graph. Figure 1 shows the hidden-node detection. The left side of the figure describes the optimal structure with observable nodes only, based on some method of the structure learning. Then, as the second step, we focus on the connections between the observable nodes shown in the right side of the figure. In this example, the parent node $x_{4}$ has the domain {1,…,5} and the child node $x_{6}$ has the one {1,…,4}. If the value of the child node is determined by only three factors, the middle node *Z*, which has the domain {1,2,3}, simplifies the conditional probability tables (CPTs). It has been known that the smaller dimension the parameter of the network is, the more accurate the parameter learning is. So, it is practically useful to find the simplest expression of the CPTs.

**Figure 2:**
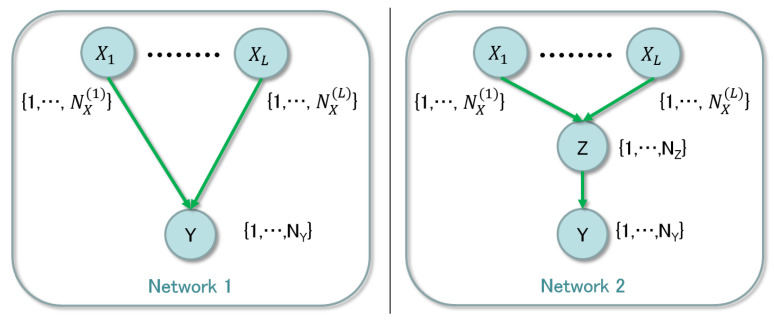
Two networks with and without a hidden node.

The issue comes down to detection of a hidden node between observable nodes. We compare two network structures, which are shown in Figure 2.

The left and the right panels are networks without and with the hidden node, respectively, where $X=(x_{1},\ldots ,x_{L})$ with the domain $x_{l}\in \left\{1,\ldots ,N_{X}^{\left(l\right)}\right\}$ and $Y\in \{1,\ldots ,N_{Y}\}$ are observable and $Z\in \{1,\ldots ,N_{Z}\}$ is hidden. Since the evidence data on *X* and *Y* are given and there is no information on *Z*, we need to consider whether the hidden node exists and its range $N_{Z}$. We propose a method to examine whether the middle hidden node should exist or not using Bayesian clustering. In order to obtain the simplest structure, there is a way to use the regularization technique [16], while it is not straightforward to prove the selected structure is theoretically optimal. Our method is justified based on a property of the entropy term in the asymptotic form of the marginal likelihood, which plays an essential role in the clustering. The result of clustering shows necessary labels to express the relation between the observable nodes *X* and *Y*. Counting the number of the used labels, we can determine the existence of the hidden node. Note that we do not consider whole possible structures of the network to reduce the computational complexity; in the present paper, we try to optimize the network from the limited structures, where for example there is no multiple inserted hidden nodes or connections between hidden nodes.

The remainder of this paper is organized as follows. Section 2 presents a formal definition of the network. Section 3 summarizes Bayesian clustering. Section 4 proposes the method to select the structure based on Bayesian clustering and derives its asymptotic behavior. Section 5 shows results of the numerical experiments validating the behavior. Finally, we present a discussion and our conclusions in Section 6 and Section 7, respectively.

## 2. Model Settings

In this section, the network structure and its parameterization are formalized. The naive structure has been applied to classification and clustering tasks and its mathematical properties are studied [5] since it is expressed as a mixture model. As mentioned in the previous section, we consider the hidden node with both parent and child observable nodes. One of the simplest networks is shown in the right panel of Figure 2. Let the probabilities of $X=(X_{1},\ldots ,X_{L})$, *Z*, and *Y* be defined by

(1) $$\begin{array}{cc} p(X_{l}=i^{\left(l\right)})= & a_{i^{\left(l\right)}}^{\left(l\right)}, \end{array}$$

(2) $$\begin{array}{cc} p(Z=j|X=i)= & b_{ij}, \end{array}$$

(3) $$\begin{array}{cc} p(Y=k|Z=j)= & c_{jk} \end{array}$$

for $i\in I=\{\left(i^{\left(1\right)},\ldots ,i^{\left(L\right)}\right)\}$, $i^{\left(l\right)}\in \left\{1,\ldots ,N_{X}^{\left(l\right)}\right\}$, $j=1,\ldots ,N_{Z}$, and $k=1,\ldots ,N_{Y}$. Since they are probabilities, we assume that

(4) $$\begin{array}{cc} a_{i}^{\left(l\right)}\geq 0, & a_{1}^{\left(l\right)}=1-\sum_{i=2}^{N_{X}^{\left(l\right)}}a_{i}^{\left(l\right)}, \end{array}$$

(5) $$\begin{array}{cc} b_{ij}\geq 0, & b_{i1}=1-\sum_{j=2}^{N_{Z}}b_{ij}, \end{array}$$

(6) $$\begin{array}{cc} c_{jk}\geq 0, & c_{j1}=1-\sum_{k=2}^{N_{Y}}c_{ij}. \end{array}$$

It is easy to find that $b_{ij}$ is the element of the CPT for *Z* and $c_{jk}$ is that for *Y*. Let *w* be the parameter consisting of $a_{i}^{\left(l\right)},b_{ij},c_{jk}$, where the dimension is

(7) $$\begin{array}{c} \dim w=\sum_{l=1}^{L}\left(N_{X}^{\left(l\right)}-1\right)+\left(N_{Z}-1\right)\prod_{l=1}^{L}N_{X}^{\left(l\right)}+N_{Z}\left(N_{Y}-1\right). \end{array}$$

We also define the probabilities of the network shown in the left panel of Figure 2;

(8) $$\begin{array}{cc} p(X^{\left(l\right)}=i^{\left(l\right)})= & d_{i^{\left(l\right)}}^{\left(l\right)}, \end{array}$$

(9) $$\begin{array}{cc} p(Y=j|X=i)= & e_{ij}. \end{array}$$

The parameter *u* consisting of $d_{i}$ and $e_{ij}$ has the dimension

(10) $$\begin{array}{c} \dim u=\sum_{l=1}^{L}\left(N_{X}^{\left(l\right)}-1\right)+\left(N_{Y}-1\right)\prod_{l=1}^{L}N_{X}^{\left(l\right)}. \end{array}$$

If the relation between *X* and *Y* can be simplified, the degree of freedom dimu is not necessary and is reduced to dimw such as the case shown in Figure 1. This is similar to the dimension reduction of data with sandglass type neural networks or the non-negative matrix factorization, which have a smaller number of nodes in the middle layers than the one in the input and output layers. The relation between the necessary dimension of the parameter and the probability of the output is not always trivial [17]. The present paper focuses on the sufficient case in terms of the dimension reduction, where dimw<dimu rewritten as

(11) $$\begin{array}{c} N_{Y}\prod_{l=1}^{L}N_{X}^{\left(l\right)}>N_{Z}\left(N_{Y}-1+\prod_{l=1}^{L}N_{X}^{\left(l\right)}\right). \end{array}$$

Recall that *X* and *Y* are observable and *Z* is hidden, where $N_{X}$ and $N_{Y}$ are given and $N_{Z}$ is unknown. When the minimum $N_{Z}$ is detected from the given evidence pairs of *X* and *Y*, and is satisfied Equation (11), the network structure with the hidden node expresses the pairs with smaller dimension of the parameter. We use Bayesian clustering technique to detect the minimum $N_{Z}$.

## 3. Bayesian Clustering

In this section, let us formally introduce Bayesian clustering. Let the evidence be described by $\left(x_{i},y_{i}\right)$ and there are *n* pairs, which are denoted by $\left(X^{n},Y^{n}\right)=\left\{\left(x_{1},y_{1}\right),\ldots ,\left(x_{n},y_{n}\right)\right\}$. Recall that $x_{i}=\left(x_{i}^{\left(1\right)},\ldots ,x_{i}^{\left(L\right)}\right)$. The corresponding value of the hidden node is $z_{i}$ and the set of *n* data is denoted by $Z^{n}$. We can estimate $z_{i}$ based on the probability $p(Z^{n}|X^{n},Y^{n})$. In Bayesian clustering, it is defined by

(12) $$\begin{array}{cc} p(Z^{n}|X^{n},Y^{n})= & \frac{p(X^{n},Z^{n},Y^{n})}{p(X^{n},Y^{n})}, \end{array}$$

(13) $$\begin{array}{cc} p(X^{n},Z^{n},Y^{n})= & \int \prod_{i=1}^{n}p\left(x_{i},z_{i},y_{i}|w\right)\varphi \left(w|\alpha \right)dw, \end{array}$$

(14) $$\begin{array}{cc} p(X^{n},Y^{n})= & \sum_{Z^{n}}p\left(X^{n},Z^{n},Y^{n}\right), \end{array}$$

where φ(w|α) is a prior distribution and α is the hyperparameter.

In the network with the hidden node,

(15) $$\begin{array}{cc} p(x_{i},z_{i},y_{i}|w)= & \prod_{l=1}^{L}\left\{a_{x_{i}^{\left(l\right)}}^{\left(l\right)}\right\}b_{x_{i}z_{i}}c_{z_{i}y_{i}}. \end{array}$$

If the prior distribution is expressed as the Dirichlet distribution for $a_{i^{\left(l\right)}}^{\left(l\right)}$, $b_{ij}$, and $c_{jk}$, the numerator $p(X^{n},Z^{n},Y^{n})$ is analytically computable. Based on the relation $p\left(Z^{n}|X^{n},Y^{n}\right)\propto p\left(X^{n},Z^{n},Y^{n}\right)$, the Markov Chain Monte Carlo (MCMC) method provides the sampling of $Z^{n}$ from $p(Z^{n}|X^{n},Y^{n})$. This is a common method to estimate hidden variables in machine learning; the underlying topics are estimated based on the Gibbs sampler in topic models such as the latent Dirichlet allocation [18].

## 4. Hidden Node Detection

In this section, the algorithm to detect the hidden node is introduced and its asymptotic property reducing the number of the used labels is revealed.

### 4.1. The Proposed Algorithm

When the size of the middle node is large such as

(16) $$\begin{array}{c} \prod_{l=1}^{L}N_{X}^{\left(l\right)}<N_{Z}, \end{array}$$

there is no reason to have the node *Z*; the middle node should reduce the degree of freedom from *X*. If only $N_{Z}=1$ satisfies Equation (11), the middle node is not necessary. Note that $N_{Z}=1$ shows that there is no edge between *X* and *Y*, which is already excluded in structure learning.

**Example** **1.** *When L=1, $N_{X}^{\left(1\right)}=3$ and $N_{Y}=3$, only $N_{Z}=1$ satisfies Equation (11), which shows that there is no hidden node between X and Y.*

The present paper proposes the following algorithm to determine the existence of *Z*;

**Algorithm** **2.** *Assume that there is $N_{Z}>1$ for given $N_{X}^{\left(l\right)}$ and $N_{Y}$, that is Equation (11) is satisfied. Apply the Bayesian clustering method to the given evidence $\left(X^{n},Y^{n}\right)$ and estimate $Z^{n}$ based on the MCMC sampling. Let the number of used labels be denoted by ${\hat{N}}_{Z}$. If the following inequality holds, the hidden node $Z\in \{1,\ldots ,{\hat{N}}_{Z}\}$ reduces the parameter,*

(17) $$\begin{array}{c} 1<{\hat{N}}_{Z}<\frac{N_{Y}\prod_{l=1}^{L}N_{X}^{\left(l\right)}}{N_{Y}-1+\prod_{l=1}^{L}N_{X}^{\left(l\right)}}. \end{array}$$

### 4.2. Asymptotic Properties of the Algorithm

The MCMC method in Bayesian clustering is based on the probability $p(X^{n},Z^{n},Y^{n})$ as shown in Section 3. Since the proposed method depends on this clustering method, let us consider the properties of $p(X^{n},Z^{n},Y^{n})$. The negative logarithm of the probability is expressed as follows:

(18) $$\begin{array}{cc} \;F_{\alpha }\left(X^{n},Z^{n},Y^{n}\right) & =-\ln p(X^{n},Z^{n},Y^{n})\\ & =-\ln\int \prod_{i=1}^{n}p\left(x_{i},z_{i},y_{i}|w\right)\varphi \left(w|\alpha \right)dw\\ & =\sum_{l=1}^{L}\left\{\ln\Gamma \left(n+N_{X}\alpha_{a}\right)-\sum_{i=1}^{N_{X}}\ln\Gamma \left(n_{i}+\alpha_{a}\right)\right\}\\ & +\sum_{i\in I}\left\{\ln\Gamma \left(\sum_{j=1}^{N_{Z}}n_{ij}+N_{Z}\alpha_{b}\right)-\sum_{j=1}^{N_{Z}}\ln\Gamma \left(n_{ij}+\alpha_{b}\right)\right\}\\ & +\sum_{j=1}^{N_{Z}}\left\{\ln\Gamma \left(\sum_{k=1}^{N_{Y}}m_{jk}+N_{Y}\alpha_{c}\right)-\sum_{k=1}^{N_{Z}}\ln\Gamma \left(m_{jk}+\alpha_{c}\right)\right\}\\ & +\sum_{l=1}^{L}\left\{N_{X}^{\left(l\right)}\ln\Gamma \left(\alpha_{a}\right)-\ln\Gamma \left(N_{X}^{\left(l\right)}\alpha_{a}\right)\right\}\\ & +\left\{\prod_{l=1}^{L}N_{X}^{\left(l\right)}\right\}\left\{N_{Z}\ln\Gamma \left(\alpha_{b}\right)-N_{X}\ln\Gamma \left(N_{Z}\alpha_{b}\right)\right\}\\ & +N_{Z}\left\{N_{Y}\ln\Gamma \left(\alpha_{c}\right)-\ln\Gamma \left(N_{Y}\alpha_{c}\right)\right\}, \end{array}$$

where $n_{i}$, $n_{ij}$, and $m_{jk}$ are given as

(19) $$\begin{array}{cc} n_{i}= & \sum_{j=1}^{n}\prod_{l=1}^{L}\delta_{x_{j}^{\left(l\right)},i^{\left(l\right)}}, \end{array}$$

(20) $$\begin{array}{cc} n_{ij}= & \sum_{k=1}^{n}\delta_{z_{k},j}\prod_{l=1}^{L}\delta_{x_{k}^{\left(l\right)},i^{\left(l\right)}}, \end{array}$$

(21) $$\begin{array}{cc} m_{jk}= & \sum_{l=1}^{n}\delta_{z_{l},j}\delta_{y_{l},k}, \end{array}$$

respectively, and the prior distribution φ(w|α) consists of the Dirichlet distributions;

(22) $$\begin{array}{cc} \varphi (w)= & \prod_{l=1}^{L}\mathrm{Dir}\left(a^{\left(l\right)}|\alpha_{a}\right)\prod_{i\in I}\mathrm{Dir}\left(b_{i}|\alpha_{b}\right)\prod_{j=1}^{N_{Z}}\mathrm{Dir}\left(c_{j}|\alpha_{c}\right), \end{array}$$

(23) $$\begin{array}{cc} \mathrm{Dir}(a^{\left(l\right)}|\alpha_{a})= & \frac{\Gamma (N_{X}^{\left(l\right)}\alpha_{a})}{\Gamma {\left(\alpha_{a}\right)}^{N_{X}^{\left(l\right)}}}\prod_{i=1}^{N_{X}^{\left(l\right)}}a_{i}^{\left(l\right)\left(\alpha_{a}-1\right)}, \end{array}$$

(24) $$\begin{array}{cc} \mathrm{Dir}(b_{i}|\alpha_{b})= & \frac{\Gamma (N_{Z}\alpha_{b})}{\Gamma {\left(\alpha_{b}\right)}^{N_{Z}}}\prod_{j=1}^{N_{Z}}b_{ij}^{\alpha_{b}-1}, \end{array}$$

(25) $$\begin{array}{cc} \mathrm{Dir}(c_{j}|\alpha_{c})= & \frac{\Gamma (N_{Y}\alpha_{c})}{\Gamma {\left(\alpha_{c}\right)}^{N_{Y}}}\prod_{k=1}^{N_{Y}}c_{jk}^{\alpha_{c}-1}. \end{array}$$

The function $\delta_{ij}$ and Γ(·) are the Kronecker delta and the gamma function, respectively. The hyperparameter α consists of $\alpha_{a}$, $\alpha_{b}$, and $\alpha_{c}$. The sampling result of $Z^{n}$ is dominantly taken from the area, which makes $p(X^{n},Z^{n},Y^{n})$ large. Then, we investigate which $Z^{n}$ minimizes $F_{\alpha }\left(X^{n},Z^{n},Y^{n}\right)$ for given $\left(X^{n},Y^{n}\right)$.

**Theorem** **3.** *When the number of the given data n is sufficiently large, $F(X^{n},Z^{n},Y^{n})$ is written as*

(26) $$\begin{array}{cc} F(X^{n},Z^{n},Y^{n})= & -nS+C\ln n+O_{p}\left(1\right), \end{array}$$

(27) $$\begin{array}{cc} S & =\sum_{l=1}^{L}\sum_{i^{\left(l\right)}=1}^{N_{X}^{\left(l\right)}}\frac{n_{i^{\left(l\right)}}^{\left(l\right)}}{n}\ln\frac{n_{i^{\left(l\right)}}^{\left(l\right)}}{n}\\ & +\sum_{i\in I}\sum_{j=1}^{{\tilde{N}}_{Z}}\frac{\sum_{j^{\prime }=1}^{{\tilde{N}}_{Z}}n_{ij^{\prime }}}{n}\frac{n_{ij}}{\sum_{j^{\prime }=1}^{{\tilde{N}}_{Z}}n_{ij^{\prime }}}\ln\frac{n_{ij}}{\sum_{j^{\prime }=1}^{{\tilde{N}}_{Z}}n_{ij^{\prime }}}\\ & +\sum_{j=1}^{{\tilde{N}}_{Z}}\sum_{k=1}^{N_{Y}}\frac{m_{j}}{n}\frac{m_{jk}}{m_{j}}\ln\frac{m_{jk}}{m_{j}}, \end{array}$$

(28) $$\begin{array}{cc} C= & \left\{\prod_{l=1}^{L}N_{X}^{\left(l\right)}\right\}\left(N_{Z}-{\tilde{N}}_{Z}\right)\alpha_{b}\\ & +\frac{1}{2}\left\{\sum_{l=1}^{L}\left(N_{X}^{\left(l\right)}-1\right)+\left(\prod_{l=1}^{L}N_{X}^{\left(l\right)}\right)\left({\tilde{N}}_{Z}-1\right)+{\tilde{N}}_{Z}\left(N_{Y}-1\right)\right\}, \end{array}$$

(29) $$\begin{array}{cc} m_{j}= & \sum_{k=1}^{N_{Y}}m_{jk}, \end{array}$$

*where ${\tilde{N}}_{Z}$ is the number of $m_{j}$ such that $m_{j}/n=O\left(1\right)$.*

The proof will be shown in Appendix A. The first term −nS is the dominant factor, and its coefficient *S* is maximized in the clustering. This coefficient determines ${\tilde{N}}_{Z}$, which is the number of used labels in the clustering result.

Assume that the true structure with the hidden node has the minimal expression, where the range of *Z* is $z=1,\ldots ,N_{Z}^{*}$, and that the estimated size is larger than the true one; $N_{Z}^{*}\leq {\tilde{N}}_{Z}$. We can easily confirm that Bayesian clustering chooses the minimum structure ${\tilde{N}}_{Z}=N_{Z}^{*}$ as follows. The three terms in the coefficient *S* correspond to the negative entropy functions of the parameter $a_{i}^{\left(l\right)}$, $b_{ij}$, and $c_{jk}$, respectively. Then, the minimum ${\tilde{N}}_{Z}$ obviously makes the coefficient *S* maximized since the number of elements of parameter should be minimized for the small entropy. When the hidden node has the redundant state, which means that two values of *Z* have completely same output distribution of *Y*, the second term of *S* is larger than the case of non-redundant situation ${\hat{N}}_{Z}=N_{Z}^{*}$. Based on the assumption that the true structure is minimal, the estimation therefore gets the minimum structure, ${\tilde{N}}_{Z}=N_{Z}^{*}$.

According to this property, the number of used label ${\hat{N}}_{Z}$ asymptotically goes to $N_{Z}^{*}$. The proposed algorithm compares the essential number of the values of *Z* and will be a criterion to select the proper structure when *n* is large. This property exists only in Bayesian clustering so far; the eliminating effect of the redundant labels has not been found in other method of the clustering such as the maximum-likelihood clustering based on the expectation-maximization algorithm.

## 5. Numerical Experiments

**Figure 3:**
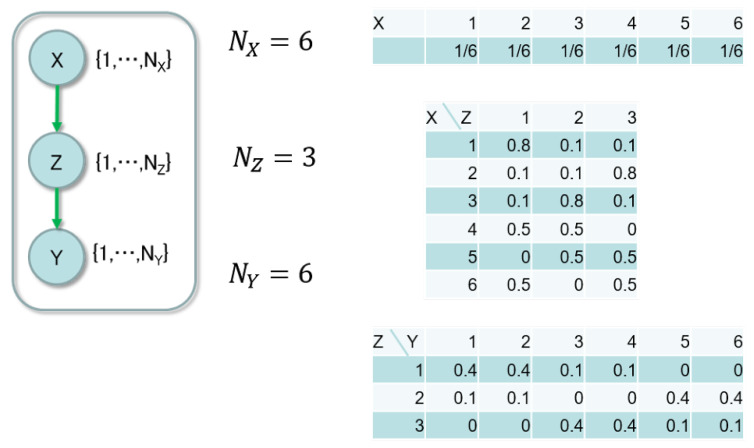
The data-generating model.

In this section, we validate the asymptotic property in numerical experiments. We set the data-generating model shown in Figure 3 and prepared ten evidence data sets.

There was a single parent node L=1. The sizes of the nodes were $N_{X}^{\left(1\right)}=6$, $N_{Y}=6$ and $N_{Z}^{*}=3$. The CPTs are described on the right-side of the figure, where the true parameter consists of these probabilities. There were 2000 pairs of (x,y) in each data set. Since the following condition is satisfied,

(30) $$\begin{array}{c} \frac{N_{Y}\prod_{l=1}^{L}N_{X}^{\left(l\right)}}{N_{Y}-1+\prod_{l=1}^{L}N_{X}^{\left(l\right)}}=\frac{6\times 6}{6-1+6}=\frac{36}{11}>3=N_{Z}^{*}, \end{array}$$

the structure of the data-generating model with the hidden node had smaller dimension of the parameter than the one without a hidden node.

We applied Bayesian clustering to each data set, where the model had the size of the hidden node $N_{Z}=6$. According to the asymptotic property in Theorem 3, the MCMC method should take label assignment from the area, where the number of the used labels was reduced to three. The estimated model size was determined by the assignment, which minimized the function $F_{\alpha }\left(X^{n},Z^{n},Y^{n}\right)$. Since the sampling of the MCMC method depended on the initial assignment, we conducted ten trials for each data set and regarded the estimated size as the minimum one. The number of iterations in the MCMC method was 1000.

Table 1 shows the results of the experiments.

In all data sets, the size of the hidden node *Z* is reduced and the correct size is estimated in more than half sets, we confirmed the effect eliminating the redundant labels. Since the result of the MCMC method depends on the given data, the minimum size is not always found; the estimated size is four in some data sets instead of three. Even in such case, however, we could estimate the correct size after setting the initial size of the model as $N_{Z}=4$. Repeating this procedure, we will be able to avoid the local optimal size and find the global one.

**Figure 4:**
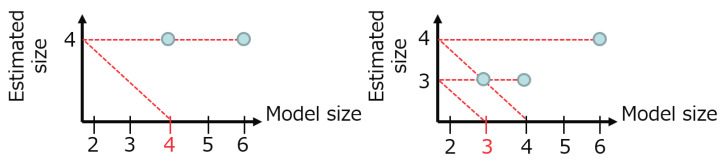
The estimation procedure in practical cases.

Figure 4 shows this estimation procedure in the practical cases. The initial model size starts from six. The left panel is the case, where the proper size is directly found and the estimated size does not change at size four. The right panel is the case, where the estimated size is first four and then the next result is three, which is the fixed point.

**Figure 5:**
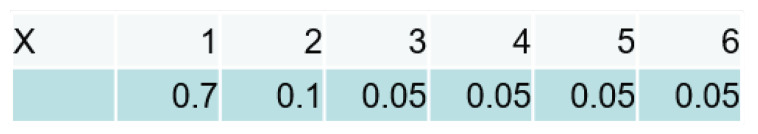
The skewed distribution of the parent node *X*.

**Figure 6:**
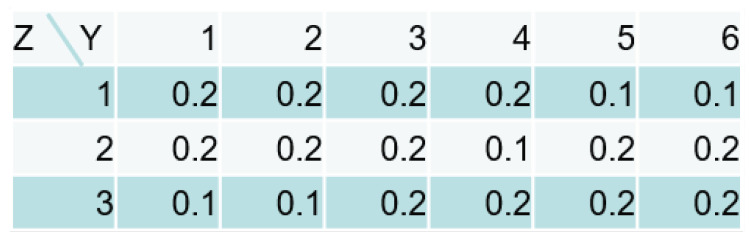
The nearly uniform distribution of the parent node *Y*.

To investigate the properties of the estimated size, we tried some different numbers of pairs n=100,500 and a skewed distribution of the parent node (Figure 5), and nearly uniform distribution of the child node (Figure 6).

Table 2 shows the results of n=100,500.

Since these CPTs of X,Z,Y are a straightforward case to distinguish the role of the hidden node, the smaller number of the pairs does not adversely affect the estimation. Table 3 shows the results of the different CPTs in the parent and the child nodes.

The number of pairs was n=100. Due to the CPT of *Z*, the skewed distribution of the parent node still keeps the sufficient variation of *Z* to estimate the size $N_{Z}$, which provides the same accuracy as the uniform distribution. On the other hand, the nearly uniform distribution of the child node makes the estimation difficult because each value of *Z* has the similar output distribution. The Dirichlet prior of *Z* has a strong effect to eliminate the redundancy, which means the estimated sizes tend to be smaller than the true one.

## 6. Discussion

In this section, we discuss the difference between the proposed method and other conventional criteria for the model selection. In the proposed method, the label assignment $Z^{n}$ is obtained from the MCMC method, which takes the samples according to $p(X^{n},Z^{n},Y^{n})$. The probability $p(X^{n},Z^{n},Y^{n})$ is the marginal likelihood on the complete data $\left(X^{n},Z^{n},Y^{n}\right)$; recall the definition,

(31) $$\begin{array}{cc} p(X^{n},Z^{n},Y^{n})= & \int \prod_{i=1}^{n}p\left(x_{i},z_{i},y_{i}|w\right)\varphi \left(w|\alpha \right)dw. \end{array}$$

This looks similar to the criteria based on the marginal likelihood such as BDu(e) [19,20] and its asymptotic form such as BIC [2], MDL [1]. Since it is assumed that the network has the statistical regularity or the nodes are all observable, many criteria do not work on the network with hidden nodes.

WBIC is proposed for the singular models. The main difference is that it is based on the marginal likelihood of the incomplete data $X^{n},Y^{n}$;

(32) $$\begin{array}{cc} \;p(X^{n},Y^{n}) & =\sum_{Z^{n}}p\left(X^{n},Z^{n},Y^{n}\right)\\ & =\int \prod_{i=1}^{n}\sum_{z_{i}}p\left(x_{i},z_{i},y_{i}|w\right)\varphi \left(w|\alpha \right)dw. \end{array}$$

Due to the marginalization over $Z^{n}$, it requires the calculation of values for all candidate structures. For example, assume that we have candidate structures $N_{Z}=1,2,3$ denoted by $p_{1}\left(X^{n},Y^{n}\right)$, $p_{2}\left(X^{n},Y^{n}\right)$, and $p_{3}\left(X^{n},Y^{n}\right)$, respectively. In WBIC, we calculate all values and select the optimal structure;

(33) $$\begin{array}{c} {\hat{N}}_{Z}=\arg\min_{i=1,2,3}p_{i}\left(X^{n},Y^{n}\right). \end{array}$$

On the other hand, in the proposed method, we calculate the label assignment with the structure $N_{Z}=3$ and obtain ${\hat{N}}_{Z}$, which shows the necessity of the node *Z*.

Another difference from the conventional criteria is the dominant order of the objective function, which determines the optimal structure. As shown in Corollary 6.1 of [6], the negative logarithm of the marginal likelihood of the incomplete data has the following asymptotic form;

(34) $$\begin{array}{cc} \;F_{\alpha }\left(X^{n},Y^{n}\right) & =-\ln p(X^{n},Y^{n})\\ & =-nS_{XY}+C_{XY}\ln n+o_{p}\left(\ln n\right), \end{array}$$

where the coefficient $S_{XY}$ is the empirical entropy of the observation $\left(X^{n},Y^{n}\right)$ and $C_{XY}$ depends on the data-generating distribution, the model, and the prior distribution. This form means that the optimal model is selected by lnn order term with the coefficient $C_{XY}$, while it is selected by *n* order term with the coefficient *S* of Theorem 3 in the proposed method. Since the largest terms are *n* order in both $F_{\alpha }\left(X^{n},Y^{n}\right)$ and $F_{\alpha }\left(X^{n},Z^{n},Y^{n}\right)$, the proposed method will have stronger effect to distinguish the difference of the structures.

The asymptotic accuracy of Bayesian clustering has been studied [21], which considers the error function between the true distribution of the label assignment and the estimated one measured by the Kullback-Leibler divergence:

(35) $$\begin{array}{cc} D(n)= & E_{X^{n},Y^{n}}\left[\sum_{Z^{n}}q\left(Z^{n}|X^{n},Y^{n}\right)\ln\frac{q(Z^{n}|X,Y^{n})}{p(Z^{n}|X^{n},Y^{n})}\right], \end{array}$$

where $E_{X^{n},Y^{n}}\left[\cdot \right]$ is the expectation over all evidence data and

(36) $$\begin{array}{cc} q(Z^{n}|X^{n},Y^{n})= & \frac{q(X^{n},Z^{n},Y^{n})}{\sum_{Z^{n}}q\left(X^{n},Z^{n},Y^{n}\right)}, \end{array}$$

(37) $$\begin{array}{cc} q(X^{n},Z^{n},Y^{n})= & \prod_{i=1}^{n}q\left(x_{i},z_{i},y_{i}\right). \end{array}$$

The true network is denoted by q(x,z,y). The proposed method minimizes this error function, which means that the label assignment $Z^{n}$ is optimized in the sense of the density estimation. Even though the optimized function is not directly for the model selection, due to the asymptotic property of the Bayes clustering simplifying the label use, the proposed method is computationally efficient to determine the existence of the hidden node and the result asymptotically has coincident.

## 7. Conclusions

In this paper, we have proposed a method to detect a hidden node between observable nodes based on Bayesian clustering. The asymptotic behavior of the clustering has been revealed and it shows that the redundant labels are eliminated and the essential structure will be detected. Evaluation of the proposed method with numerical experiments is one of our future studies.

## Figures and Tables

**Table 1:** The results of the estimated size.

Data-Set ID	1	2	3	4	5	6	7	8	9	10
Estimated size	3	3	3	4	3	3	4	4	4	3

**Table 2:** The results of the estimated size in n=100,500.

Data-Set ID	1	2	3	4	5	6	7	8	9	10
Estimated size (*n* = 100)	3	3	3	3	4	3	4	3	3	3
Estimated size (*n* = 500)	3	3	3	3	3	3	3	3	3	4

**Table 3:** The results of the estimated size in the different conditional probability tables (CPTs).

Data-Set ID	1	2	3	4	5	6	7	8	9	10
Estimated size (skewed parent node)	3	3	3	3	3	4	3	3	3	3
Estimated size (nearly-uniform child node)	1	1	1	1	2	1	1	2	1	1

## Appendix A

In this section, we prove Theorem 3. Using the asymptotic relation $\ln\Gamma \left(x\right)=x\ln x-\frac{1}{2}\ln x-x+O\left(1\right)$ for sufficiently large *x*, we can obtain that

(A1) $$\begin{array}{cc} F_{\alpha }\left(X^{n},Z^{n},Y^{n}\right) & =\sum_{l=1}^{L}\{\left(n+N_{X}^{\left(l\right)}\alpha_{a}\right)\ln\left(n+N_{X}^{\left(l\right)}\alpha_{a}\right)\\ & -\frac{1}{2}\ln\left(n+N_{X}^{\left(l\right)}\alpha_{a}\right)-\left(n+N_{X}^{\left(l\right)}\alpha_{a}\right)\}\\ & -\sum_{l=1}^{L}\sum_{i^{\left(l\right)}=1}^{N_{X}^{\left(l\right)}}\{\left(n_{i^{\left(l\right)}}^{\left(l\right)}+\alpha_{a}\right)\ln\left(n_{i^{\left(l\right)}}^{\left(l\right)}+\alpha_{a}\right)\\ & -\frac{1}{2}\ln\left(n_{i^{\left(l\right)}}^{\left(l\right)}+\alpha_{a}\right)-\left(n_{i^{\left(l\right)}}^{\left(l\right)}+\alpha_{a}\right)\}\\ & +\sum_{i\in I}\{\left(\sum_{j=1}^{N_{Z}}n_{ij}+N_{Z}\alpha_{b}\right)\ln\left(\sum_{j=1}^{N_{Z}}n_{ij}+N_{Z}\alpha_{b}\right)\\ & -\frac{1}{2}\ln\left(\sum_{j=1}^{N_{Z}}n_{ij}+N_{Z}\alpha_{b}\right)-\left(\sum_{j=1}^{N_{Z}}n_{ij}+N_{Z}\alpha_{b}\right)\}\\ & -\sum_{i\in I}\sum_{j=1}^{N_{Z}}\left\{\left(n_{ij}+\alpha_{b}\right)\ln\left(n_{ij}+\alpha_{b}\right)-\frac{1}{2}\ln\left(n_{ij}+\alpha_{b}\right)-\left(n_{ij}+\alpha_{b}\right)\right\}\\ & +\sum_{j=1}^{N_{Z}}\{\left(\sum_{k=1}^{N_{Y}}m_{jk}+N_{Y}\alpha_{c}\right)\ln\left(\sum_{j=1}^{N_{Y}}m_{jk}+N_{Y}\alpha_{c}\right)\\ & -\frac{1}{2}\ln\left(\sum_{k=1}^{N_{Y}}m_{jk}+N_{Y}\alpha_{c}\right)-\left(\sum_{k=1}^{N_{Y}}m_{jk}+N_{Y}\alpha_{c}\right)\}\\ & -\sum_{j=1}^{N_{Z}}\sum_{k=1}^{N_{Y}}\left\{\left(m_{jk}+\alpha_{c}\right)\ln\left(m_{jk}+\alpha_{c}\right)-\frac{1}{2}\ln\left(m_{jk}+\alpha_{c}\right)-\left(m_{jk}+\alpha_{c}\right)\right\}\\ & +O_{p}\left(1\right). \end{array}$$

Collecting the constant terms and uniting them to $O_{p}\left(1\right)$, we rewrite $F_{\alpha }\left(X^{n},Z^{n},Y^{n}\right)$ as

(A2) $$\begin{array}{cc} F_{\alpha }\left(X^{n},Z^{n},Y^{n}\right) & =\sum_{l=1}^{L}\left\{n\ln n+N_{X}^{\left(l\right)}\alpha_{a}\ln n-\frac{1}{2}\ln n-n\right\}\\ & -\sum_{l=1}^{L}\sum_{i^{\left(l\right)}=1}^{N_{X}^{\left(l\right)}}\left\{n_{i^{\left(l\right)}}^{\left(l\right)}\ln n_{i^{\left(l\right)}}^{\left(l\right)}+\alpha_{a}\ln n_{i^{\left(l\right)}}^{\left(l\right)}-\frac{1}{2}\ln n_{i^{\left(l\right)}}^{\left(l\right)}-n_{i^{\left(l\right)}}^{\left(l\right)}\right\}\\ & +\sum_{i\in I}\left\{\left(\sum_{j=1}^{N_{Y}}n_{ij}\right)\ln\sum_{j=1}^{N_{Y}}n_{ij}+N_{Z}\alpha_{b}\ln\sum_{j=1}^{N_{Z}}n_{ij}-\frac{1}{2}\ln\sum_{j=1}^{N_{Z}}n_{ij}-\sum_{j=1}^{N_{Z}}n_{ij}\right\}\\ & -\sum_{i\in I}\sum_{j=1}^{N_{Z}}\left\{n_{ij}\ln n_{ij}+\alpha_{b}\ln n_{ij}-\frac{1}{2}\ln n_{ij}-n_{ij}\right\}\\ & +\sum_{j=1}^{N_{Z}}\{\sum_{k=1}^{N_{Y}}m_{jk}\ln\left(\sum_{k=1}^{N_{Y}}m_{jk}\right)+N_{Y}\alpha_{b}\ln\sum_{k=1}^{N_{Y}}m_{jk}\\ & -\frac{1}{2}\ln\sum_{k=1}^{N_{Y}}m_{jk}-\sum_{k=1}^{N_{Y}}m_{jk}\}\\ & -\sum_{j=1}^{N_{Z}}\sum_{k=1}^{N_{Y}}\left\{m_{jk}\ln m_{jk}+\alpha_{c}\ln m_{jk}-\frac{1}{2}\ln m_{jk}-m_{jk}\right\}+O_{p}\left(1\right). \end{array}$$

Using the following relations,

(A3) $$\begin{array}{cc} \sum_{i^{\left(l\right)}=1}^{N_{X}^{\left(l\right)}}n_{i^{\left(l\right)}}^{\left(l\right)}\ln n_{i^{\left(l\right)}}^{\left(l\right)}= & n\sum_{i^{\left(l\right)}=1}^{N_{X}^{\left(l\right)}}\left(\frac{n_{i^{\left(l\right)}}^{\left(l\right)}}{n}\ln\frac{n_{i^{\left(l\right)}}^{\left(l\right)}}{n}\right)+n\ln n, \end{array}$$

(A4) $$\begin{array}{cc} \sum_{i\in I}\left(\sum_{j=1}^{N_{Z}}n_{ij}\right)\ln\sum_{j=1}^{N_{Z}}n_{ij}= & n\sum_{i\in I}\left(\frac{n_{i}}{n}\ln\frac{n_{i}}{n}\right)+n\ln n, \end{array}$$

(A5) $$\begin{array}{cc} \sum_{i\in I}\sum_{j=1}^{N_{Z}}n_{ij}\ln n_{ij}= & n\sum_{i\in I}\sum_{j=1}^{N_{Z}}\frac{n_{ij}}{n}\ln\frac{n_{ij}}{n}+n\ln n, \end{array}$$

(A6) $$\begin{array}{cc} \sum_{j=1}^{N_{Z}}\left(\sum_{k=1}^{N_{Y}}m_{jk}\right)\ln\sum_{k=1}^{N_{Y}}m_{jk}= & n\sum_{j=1}^{N_{Z}}\frac{m_{j}}{n}\ln\frac{m_{j}}{n}+n\ln n, \end{array}$$

(A7) $$\begin{array}{cc} \sum_{j=1}^{N_{Z}}\sum_{k=1}^{N_{Y}}m_{jk}\ln m_{jk}= & n\sum_{j=1}^{N_{Z}}\sum_{k=1}^{N_{Y}}\frac{m_{jk}}{n}\ln\frac{m_{jk}}{n}+n\ln n \end{array}$$

and focusing on the terms of order *n* and lnn, we obtain the asymptotic form in the theorem.
